# The Volar Base of the Third Metacarpal Is Not a Palpable Landmark in the Palm

**DOI:** 10.7759/cureus.94732

**Published:** 2025-10-16

**Authors:** Andrew Boone, Taylor Paskey, Robert J Strauch

**Affiliations:** 1 Orthopedic Surgery, Columbia University College of Physicians and Surgeons, New York, USA

**Keywords:** cadaveric study, carpal tunnel, median nerve anatomy, thenar branch localization, transverse carpal ligament

## Abstract

Various techniques have been described to identify the topographical projection of the thenar branch of the median nerve (TBMN). This study explores two hypotheses: (1) There is a palpable landmark overlying the volar base of the third metacarpal, and (2) this landmark is the transverse carpal ligament, not the third metacarpal base itself. Using four upper extremities from two cadavers, manual palpation was performed to identify and mark the palpable landmark overlying the volar base of the third metacarpal using a radiopaque marker. The distance between the palpable landmark and the volar base of the third metacarpal was measured using fluoroscopy. Anatomic dissection was then performed to identify the basis of the landmark. The palpable landmark was confirmed to be the distal end of the transverse carpal ligament in all specimens. Therefore, there is a palpable landmark beneath the palmar skin overlying the volar base of the third metacarpal, and it is the distal edge of the transverse carpal ligament, not the metacarpal base.

## Introduction

Various techniques have been described to identify the topographical projection of the thenar branch of the median nerve (TBMN). Some studies have described intersecting lines from landmarks of the hand to locate the TBMN [1-4]. The middle finger flexion test has also been reported to localize the TBMN [5]. Recently, Bertelli et al. described palmar palpation of the volar base of the third metacarpal as a landmark for locating the TBMN [6]. The third metacarpal base was not previously described as an anatomic landmark that can be palpated through the volar skin of the palm. The volar base of the third metacarpal, however, is relatively shielded from palpation by the flexor tendons and the transverse carpal ligament. This study explores two hypotheses: (1) There is a palpable landmark overlying the volar base of the third metacarpal, and (2) the palpable landmark overlying the volar base of the third metacarpal is the transverse carpal ligament, not the volar base of the third metacarpal.

## Case presentation

**Figure 1:**
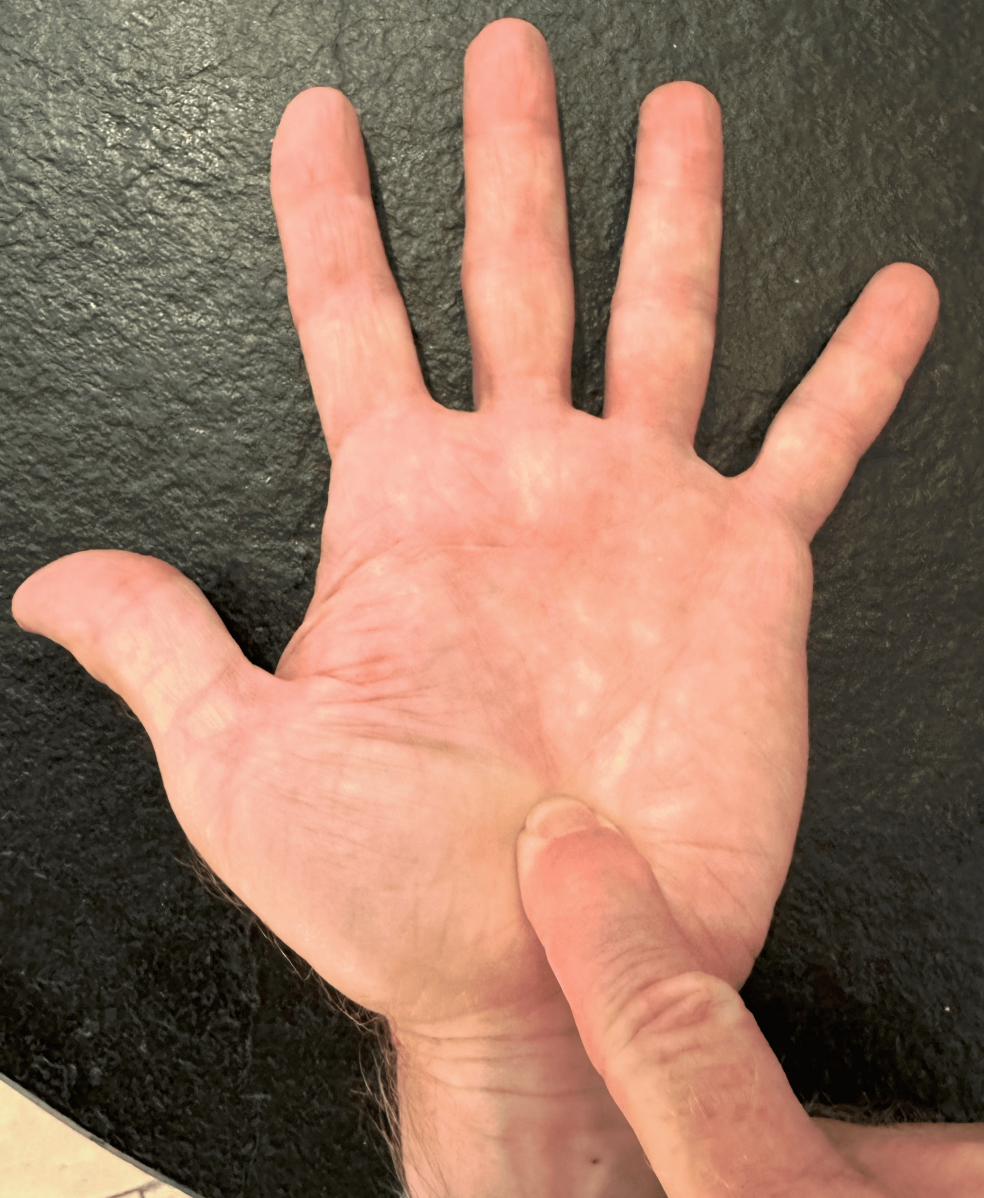
Volar palpation test for the base of the third metacarpal.

Four upper extremities were obtained, providing two left and two right wrists. Two specimens were from a 57-year-old man, and the other two specimens were from a 67-year-old woman. The palm was manually palpated by the two senior investigators starting from the distal palmar crease and going proximally, in line with the third metacarpal, until a discrete palpable firmness was appreciable (Figure 1). This location was termed the “palpable landmark.”

**Figure 2:**
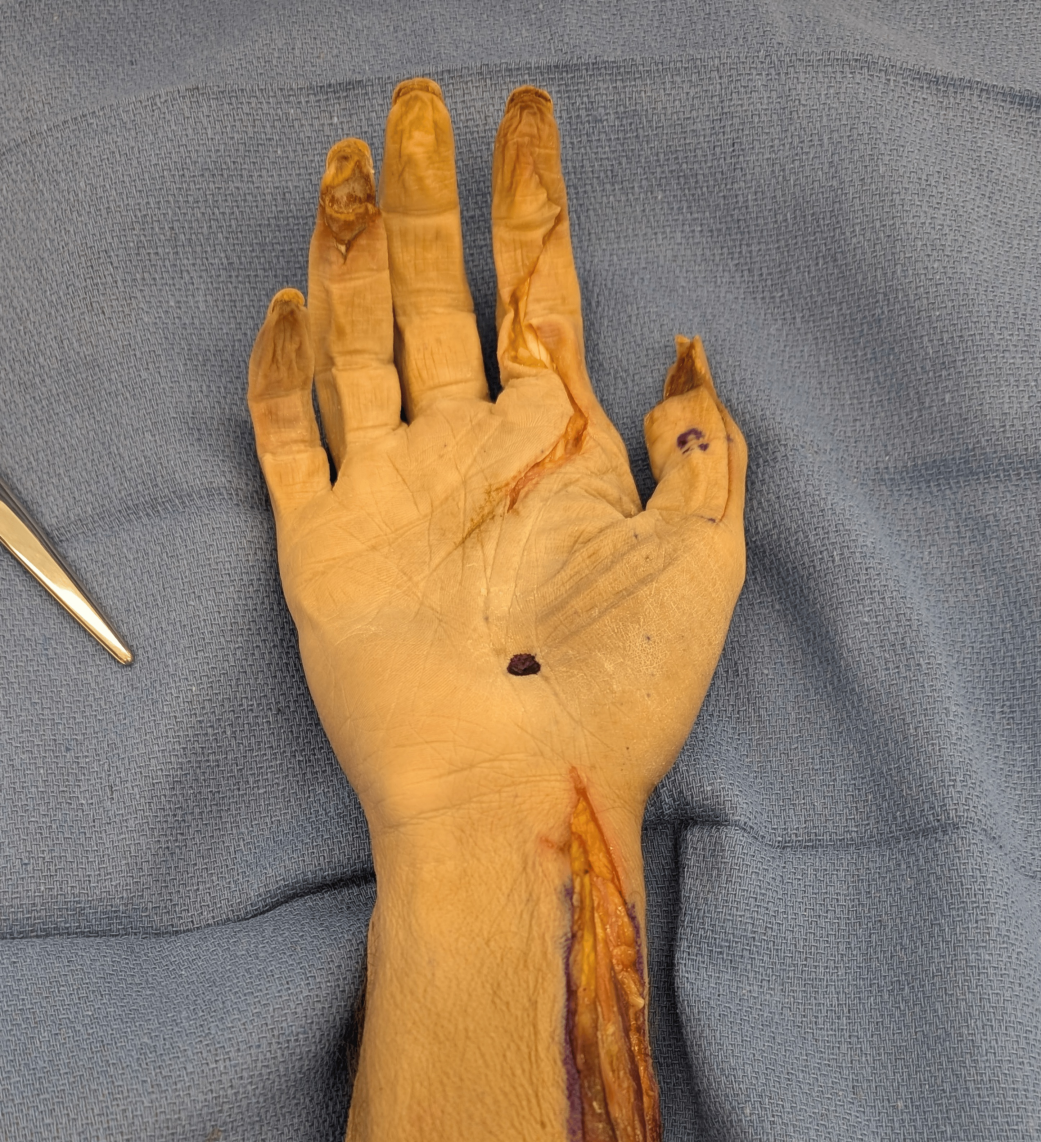
Volar palpation was attempted in the palm and marked. This location was then evaluated on fluoroscopy.

**Figure 3:**
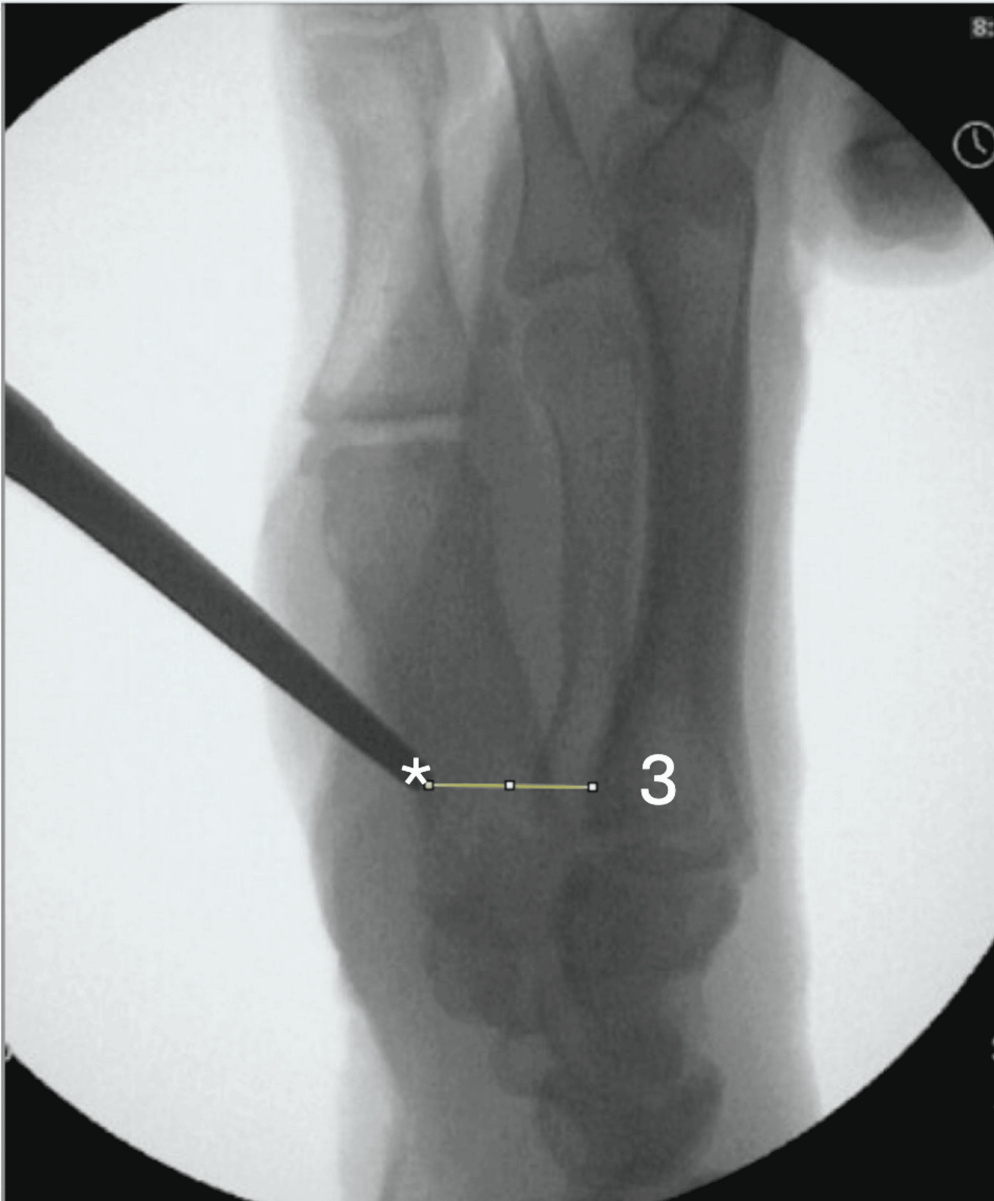
After the palpable landmark was identified and the fluoroscopic image was obtained, the distance between the anatomic base of the third metacarpal and the tip of the metallic instrument on the lateral view was measured using ImageJ (National Institutes of Health, Bethesda, MD). 3 = the base of the third metacarpal; * = the tip of the metallic probe that represents the palpable landmark.

**Figure 4:**
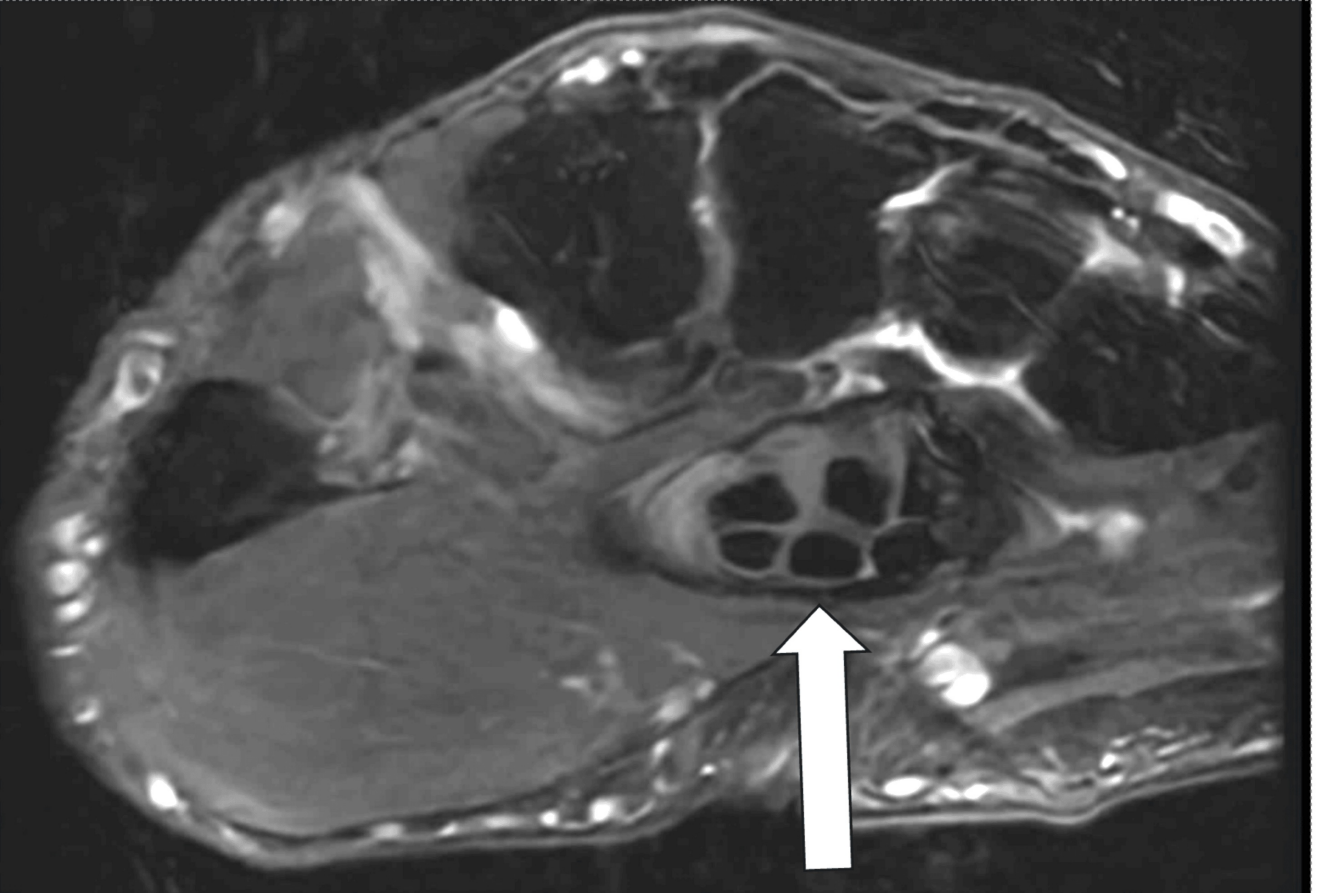
Axial T2 MRI of the wrist at the level of the third metacarpal base that demonstrates the palpable landmark (the distal edge of the transverse carpal ligament) in relation to the third metacarpal base. White arrow = distal edge of the transverse carpal ligament.

**Figure 5:**
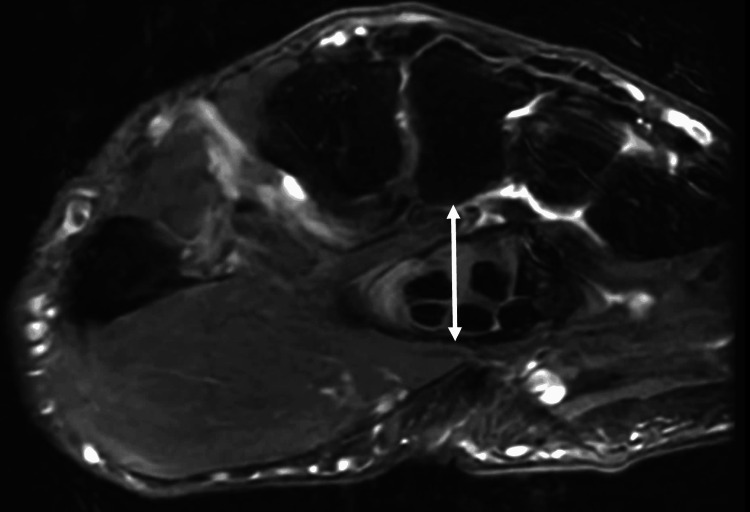
Axial T2 MRI of the wrist at the level of the third metacarpal base that demonstrates the distance between the palpable landmark (the distal edge of the transverse carpal ligament) and the volar base of the third metacarpal. This distance, which is denoted by the double-headed white arrow on the MRI, measured 1.6 cm.

The location of the palpable landmark was marked (Figure 2), and a radiopaque steel instrument (Mayo scissors, Mayo Clinic, Rochester, MN) was then placed directly over the landmark and depressed into the skin. Mini-fluoroscopy was then used to measure the distance between the instrument and the volar base of the third metacarpal on lateral films (Figure 3). The instrument was removed, and an extended carpal tunnel incision was made. The anatomic basis for the palpable landmark was then examined once the palmar fascia was divided. The transverse carpal ligament was then incised longitudinally, and the median nerve and the flexor tendons were retracted to expose the underlying third metacarpal base (Figures 4, 5).

Upon the visualization and direct palpation of the metacarpal base, we obtained repeat fluoroscopic images with the instrument in contact with the third metacarpal base, confirming the position of the volar tuberosity. The fluoroscopic images were uploaded to the ImageJ software. The measurements were scaled and calibrated to millimeters. On the lateral views, the shortest distance from the tip of the radiopaque instrument prior to skin incision to the most volar aspect of the base of the third metacarpal was determined.

During the initial attempted palpation of the volar tuberosity of the third metacarpal on the cadavers, prior to any incision, all specimens demonstrated the presence of a palpable landmark. Upon dissection down to the palpable landmark, it was clear that in all specimens the palpable landmark consisted of the distal end of the transverse carpal ligament and not the volar tuberosity of the third metacarpal base. Following the transection of the transverse carpal ligament, the palpable landmark disappeared and was no longer palpable. Lateral fluoroscopic images confirmed that it was impossible to directly palpate the volar third metacarpal base. The palpable landmark was on average about 10.4 mm volar to the volar tuberosity of the third metacarpal base (Table 1).

**Table 1:** Distance between the palpable landmark identified by the radiopaque instrument and the base of the third metacarpal on fluoroscopy.

Distance From the Palpable Landmark to the Volar Tuberosity on Lateral Imaging	
Cadaver 1, Right Arm	15.0 mm
Cadaver 1, Left Arm	9.46 mm
Cadaver 2, Right Arm	7.67 mm
Cadaver 2, Left Arm	9.41 mm
Average	10.4 mm
Standard Deviation	3.19 mm

## Discussion

Various studies have attempted to localize the TBMN based upon anatomic landmarks. The middle finger flexion test, proposed by Rodriguez and Strauch, identified the motor branch approximately 0.9 mm proximal and 1.9 mm ulnar to the tip of the middle finger when the proximal interphalangeal joint and metacarpophalangeal joint were each passively brought into 90 degrees of flexion with the distal interphalangeal joint extended [5]. Wilhelmi et al. found that the TBMN was 6.3 mm proximal and 8.6 mm radial from the intersection of two lines: one line connecting the hook of hamate and the radial aspect of the metacarpophalangeal crease of the thumb and the other line connecting the second web space and scaphoid tubercle [7]. Eskandari et al. placed the TBMN 12.6 mm ulnar and 4.4 mm proximal to the intersection of Kaplan’s cardinal line, originally described in 1953 as a line from the interdigital fold of the thumb and index to the ulnar aspect of the hand and parallel to the middle crease, and the radial border of the third ray [8].

All of these studies are estimates of the location of the nerve, taking into account anatomic variations of the thenar branch. Kozin emphasized that the most common variation (74%) is the motor branch coming off the median nerve distal to the transverse carpal ligament, surrounded by some investing oblique fascial fibers communicating with the overlying palmar aponeurosis [2]. This is in comparison to the nerve branching distal but surrounded by only muscle (19%) and the least common variation being the true transligamentous subtype (7%). His study also denoted that nearly all the motor branches in the 101 cadavers had a takeoff that was central volar or just radial to this central position on the median nerve, only one directly radial and no branches ulnar [2].

Bertelli et al.’s study describing the volar palpation of the third metacarpal base showed excellent agreement between various observers. However, there were no lateral radiographs of the third metacarpal in Bertelli et al.’s study, only AP radiographs. Therefore, it was impossible to determine that the observers were palpating the third metacarpal base. Had lateral radiographs been obtained, it likely would have been clear that the palpated finger was not abutting the third metacarpal base [6]. When the palm is palpated from distal to proximal in line with the third metacarpal, there is a palpable landmark that overlies the AP projection of the third metacarpal base. This landmark is not the third metacarpal base itself; rather, it is the distal edge of the transverse carpal ligament.

## Conclusions

In conclusion, this study has found that there is a palpable landmark overlying the volar base of the third metacarpal and that this landmark is the distal edge of the transverse carpal ligament. When the ligament is incised, the landmark disappears. Therefore, the third metacarpal base itself is not palpable through the skin as it lies a centimeter beneath the transverse carpal ligament and the flexor tendons. One limitation of this study is the low sample size of cadavers. However, we do not believe that a larger sample size would affect our conclusion, since anatomy is relatively consistent between individuals; i.e., the transverse carpal ligament and flexor tendons always cover the volar base of the third metacarpal. This investigation refutes prior literature that the volar base of the third metacarpal is palpable in the palm.
